# Gender Gap in Neurology Research Authorship (1946–2020)

**DOI:** 10.3389/fneur.2021.715428

**Published:** 2021-08-23

**Authors:** Anne X. Nguyen, Lilian Yoffe, Anna Li, Xuan-Vi Trinh, Jerry Kurian, Heather E. Moss, Albert Y. Wu

**Affiliations:** ^1^Faculty of Medicine, McGill University, Montreal, QC, Canada; ^2^Department of Physiology, McGill University, Montreal, QC, Canada; ^3^Faculty of Science, McGill University, Montreal, QC, Canada; ^4^Department of Computer Science, McGill University, Montreal, QC, Canada; ^5^Department of Ophthalmology, Stanford University School of Medicine, Palo Alto, CA, United States; ^6^Department of Neurology & Neurological Science, Stanford University School of Medicine, Palo Alto, CA, United States

**Keywords:** gender, equity, neurology, disparity, authorship

## Abstract

Gender disparity in the field of neurology impedes scientific advancements and innovations. In 2018, 45.0% of neurology and neurological subspecialty residents were women. Despite a notable rise in the proportion of women neurologists over the past decades, inequalities regarding publication proportions between men and women persist in the field. This cohort study examines authorship trends in articles published in 155 international neurology journals, identified as those listed in the annual Journal Citation Reports' “Clinical Neurology” section. Authors' names, authorship positions and countries of affiliation were extracted from PubMed for indexed articles published from 1946 to 2020. Gender-API (a validated and highly accurate application program interface) assigned binary genders to authors. Author gender proportions were compared across subspecialties, authorship position and years. In 303,385 unique articles, 1,663,036 total authors were identified of which 34.1% were women. Neuroradiology demonstrated the lowest proportion of women authors (21.3%), while neurogenetics displayed the highest (44.5%). In articles with multiple authors, both men and women last authors were more likely to publish with a male first author, though this was significantly more pronounced for men last authors (1.86 vs. 1.08; *p* < 0.001). From 2002 to 2020, women remained in the minority of last (24.6%), first (36.2%), and middle author positions (35.8%). The authorship gender distribution in neurological journals neither reflects the gender proportion of neurologists in the field overall nor in any subspecialty examined. We also find a tendency for senior and junior authors of the same gender to publish together which perpetuates authorship inequity. Further work is needed to identify underlying causes so that interventions might be developed to improve authorship diversity.

## Introduction

While the numbers of women researchers and clinicians in medicine are increasing, a significant gender gap in academic neurology persists (1). Gender disparity remains in higher faculty ranks (25.0% of full professors in 2018 were women) even though the proportions of women residents in neurology and neurological subspecialties (45.0% in 2018) are approaching the ratio of women medical school graduates (47.9% in 2018–2019) (2). These unequal proportions may result from gender biases that are barriers to women neurologists obtaining funding, authorship and recognition, as well as attaining leadership positions (1). For instance, neurology has one of the largest gender pay gap among all the medical fields (3).

Improving gender and cultural diversity in perspective is key to promote scientific advancements and to improve the quality of research (4). While the contributions of individuals coming from minority groups are often disregarded, their outlook can greatly benefit collaborative efficacy and refine the quality of reasoning given that they provide a new way of thinking (4). A study has shown that a more diversified group tend to produce a larger variety of approaches and solutions to problems (5). Others have indicated that promoting sex diversity improved scientific rigor through teamwork among academics (6). Another paper has suggested that increasing the number of individuals from underrepresented groups in the workforce can help diminish the discriminatory barriers felt by those individuals which ultimately strengthens a work group's effectiveness (7).

This bibliometric study attempts to analyze authorship gender trends in neurology publications. It builds on a prior study that highlighted the lack of gender parity seen in authorship in three high impact American neurology journals over 35 years (1980–2015) (8). This paper shows an increase over time in first authorship position for women from 7.9 to 24.6% and in last authorship position from 6.1 to 18.1% (8). Though encouraging, this growth did not match the proportions of women neurology residents (48.4%) and faculty (37.0%) in 2015 (2). Authorship positions (first, middle, last) are suggestive of the authors' contribution and their academic standings. Thus, the gender disparity within these positions suggests that barriers to advancement exist in neurology academia.

Gender parity in authorship is important not only as a marker of scientific parity and achievement since publishing is crucial in all stages of academic career advancement, but also as a marker of the diversity of perspectives in the literature (9–11). This study highlights past and existing gender disparity in authorship in neurology and related field journals, quantifying a disparity at each authorship position, and in all neurology subspecialties. Although the analysis over a 74-year period demonstrated increasing proportions of women authors over time, women authorship proportions remained disproportionately low when compared to the increasing rate of women neurologists (2). The existing literature suggest that the largest gender disparity is in last authorship position (12, 13) which is concerning as this may be a marker of senior leadership in the field.

Further analyses of authorship gender disparity in neurology and neurology-related fields are needed to better understand the situation and to address authorship inequity. To date, no report has assessed longitudinal gender authorship disparity encompassing the international neurology literature, spanning over 74 years. This study examines the authorship gender distribution per authorship position, journal category (subspecialty) and journal-level metric. Author gender of within-article authorship collaborations is also examined.

## Materials and Methods

### Journal Selection

**Figure 1:**
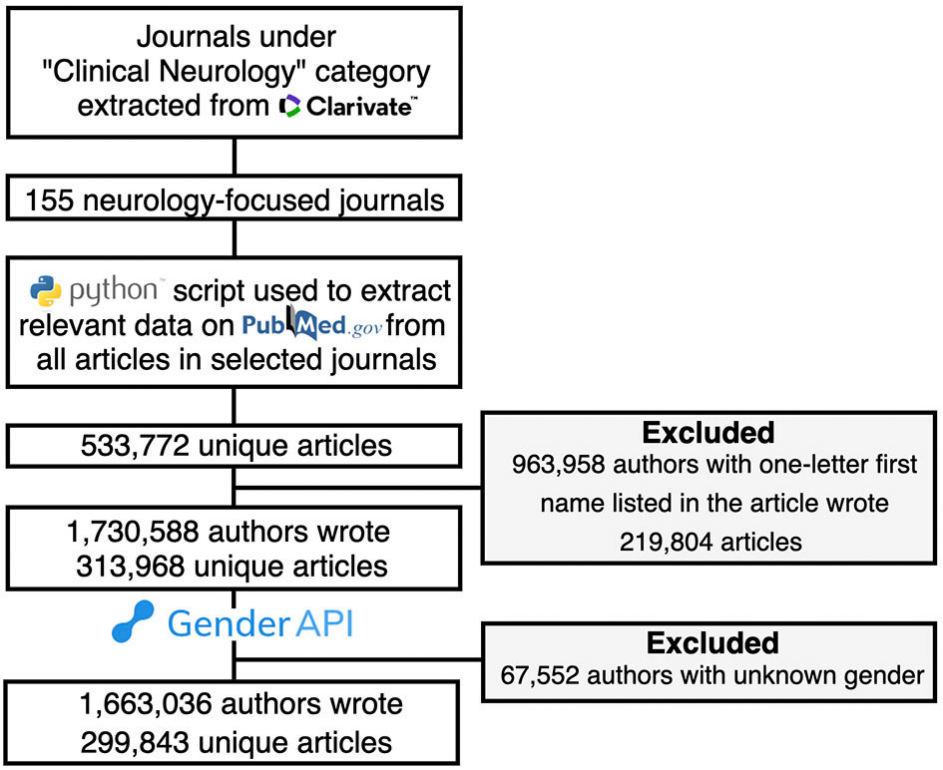
Study flowchart. The logos are from the company websites: Clarivate (https://clarivate.com/), Python (https://www.python.org/), PubMed (https://pubmed.ncbi.nlm.nih.gov/) and Gender API (https://gender-api.com/).

All neurology-related journals (excluding journals focused on neurosurgery, orthopedics, psychiatry, and allied health fields) listed in the “Clinical Neurology” category of Clarivate Analytics' InCites Journal Citation Reports (JCRs) (jcr.clarivate.com) from 1997 to 2019 were included (Figure 1). Journal-level metrics (Impact Factor, EigenFactor, Total Cites) of all neurology journals are collected as measures to find potential correlations between the prestige and the authorship distribution. Relevant to this, journal information from the 2019 JCR was recorded: Total Cites (total number of times a journal has been cited in 2019) (14), the Journal Impact Factor (score based on the average number of citations articles in a given journal receive during 2017 to 2018 year) (15), and the Eigenfactor score (score based on the number of times articles published from 2015 to 2019) (16).

### Journal Category

Three authors (AN, LY, AL) classified each journal into one or more of 24 subspecialties (neurology-related categories) using the National Library of Medicine (NLM) Catalog's Medical Subject Headings and Broad Subject Terms as references. When consensus between all three authors was not attained, subspecialty distinction was made based on the title and journal description. The journal categories were subsequently revised by experts in the field (HM, AW).

As some journals were categorized into multiple subspecialties, a scoring system was used to generate an accurate gender proportion per category and to ensure a fair distribution among all categories. More specifically, the number of authors per gender for each journal was divided by the number of categories associated with that journal. For example, if a journal belonged to two different categories (e.g., neurophysiology and neuroradiology), half the number of women authors and men authors from that journal counted toward each category. These scores were generated for all journals and summed appropriately to generate a total author count per gender for each category.

### Dataset Characteristics

A script was performed to extract all relevant data of PubMed literature (1946–2020) from the selected journals on January 15th, 2021. PubMed is a database housed in the National Library of Medicine (NLM) and the publications extracted encompass all types of documents that are searchable by a query on PubMed (e.g., articles, commentaries, editorials). NLM underwent a reinvention in the early 2000s, which concurs with the substantial increase of literature indexed in PubMed at that time (17). The number of authors per year therefore depends on PubMed's indexing system. To address this feature, we further analyzed the PubMed publications and authors based on two distinct periods: 1946–2001 (low number of authors per year, specifically <1,000 authors annually), and 2002–2020 (significant rise in the number of authors per year starting from 2002 coinciding with the NLM reinvention).

The script removed duplicates from the dataset based on publication titles. The extracted information included the title, year published, PubMed Central Identifier (PMCID, a standard identifier for each publication in PMC), and author information (first name, last name, affiliated institution). Authorship position by publication was determined as follows: sole authors or the first listed author was “first author,” the last listed author was “last author,” and authors in between were “middle author(s).”

### Author Gender Assignment

Each author's first name and country of their affiliated institution (if known) were input to Gender-API (https://gender-api.com/) to determine their gender. Gender-API is an application program interface (API) used to classify a person's gender based on their first name and, optionally, their location, for improved accuracy. This proprietary algorithm is built upon combined data from several sources including data from social networks and governmental records that are publicly available (18). It has been proven to be the most accurate gender assignment program with an accuracy exceeding 98.0% (18). It returns the person's gender (man, woman or undetermined), the assignment's accuracy (in percentage) and the sample size (number of records found in Gender-API's database which matches the given input). Authors whose gender was undetermined were excluded from the dataset while the remaining authors whose gender could be determined from the same article were included in the dataset. Despite the use of the term “gender” in this study, it is important to note that it is derived from the author's first name through the use of gender-API.

### Statistical Analysis

The data was analyzed with STATA/SE version 16.1 (Stata Corp, College Station, Texas, USA). The main outcome examined was authorship gender. Pearson's Chi-Square test was performed to evaluate whether there was a significant relationship between authorship gender and authorship position overall. Cuzick non-parametric tests for trend were performed to measure the difference between men and women author proportions by authorship position over time. Spearman correlation was calculated to analyze the relationship between gender proportion and time (in years). Pearson's Chi-Square test was used to assess gender differences among all the 24 journal categories at once. Spearman's correlation (rho) was calculated to analyze the relationship between authorship gender proportions and the journal-level metrics (Impact Factor, Eigenfactor, Total Cites), all of which showed non-normal distributions. McNemar's test was used to analyze the relationship between the gender of last authors and the gender of first authors within articles. *P* ≤ 0.05 were considered statistically significant.

## Results

A total of 1,730,588 authors were extracted from 299,843 articles in 155 neurology journals (Figure 1). Gender-API assigned the gender of 96.1% (*n* = 1,663,036) authors with a mean accuracy of 94.6% and a standard deviation of 10.3%. More specifically, 1,331 out of 290,542 first author genders along with 4,144 out of 1,120,512 middle author genders and 745 out of 258,211 last author genders were not successfully classified by Gender-API. One hundred fifty-two thousand four hundred seventy articles had both first and last author with assigned genders. Examples of authors with an undetermined gender included group authors (e.g., The Alzheimer's Disease Neuroimaging Initiative), single named authors (e.g., Itti), and authors with incomplete first names (e.g., Edwards, D.). Out of 1,663,036 gendered authors, 34.1% (*n* = 567,904) were women.

### Gender Distribution per Authorship Position

**Figure 2:**
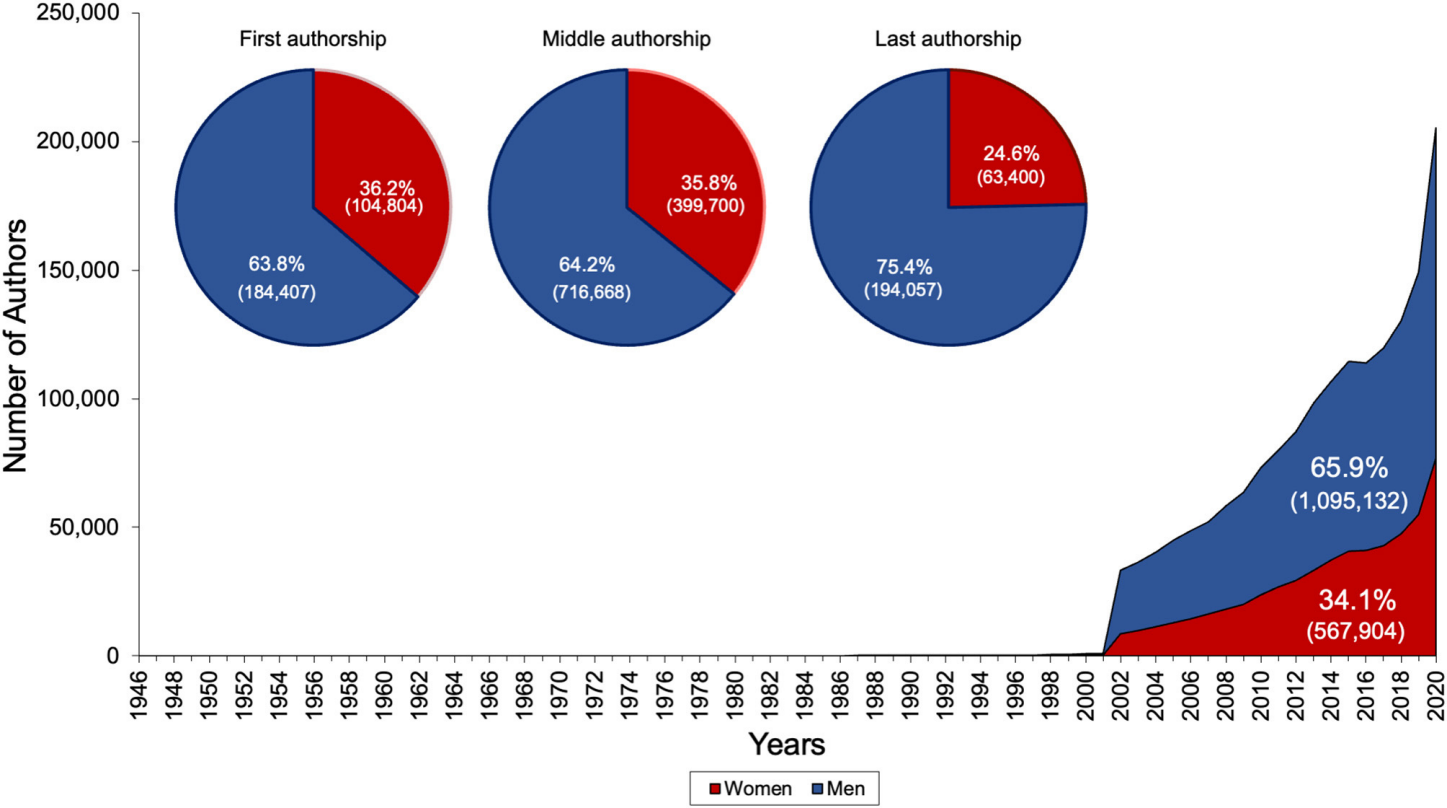
Overall gender authorship distribution, with pie charts representing the gender authorship distribution overall and per authorship position (first, middle, last).

Throughout all years studied, gender and authorship position were associated (chi-square, *P* < 0.001). The smallest proportion of women authors was among last authors (24.6%; 63,400/257,457 women), followed by middle authors (35.8%; 399,700/1,116,368 women) and then first authors (36.2%; 104,804/289,211 women) (Figure 2).

### Gender Authorship Distribution per Time Period

During the years prior to the NLM reinvention, between 1946 and 2001, 13.7% [894/6,549 (0 to 184/year)] of authors were women. Women occupied 15.1% (284/1,887) of first author positions, 14.1% (416/2,942) of middle author positions, and 11.3% (194/1,720) of last author positions. There was a weak correlation between gender proportion and year (Spearman rho = 0.50, Cuzick *P* < 0.001). Sub-analyses by authorship position were not performed due to the small sample size.

**Figure 3:**
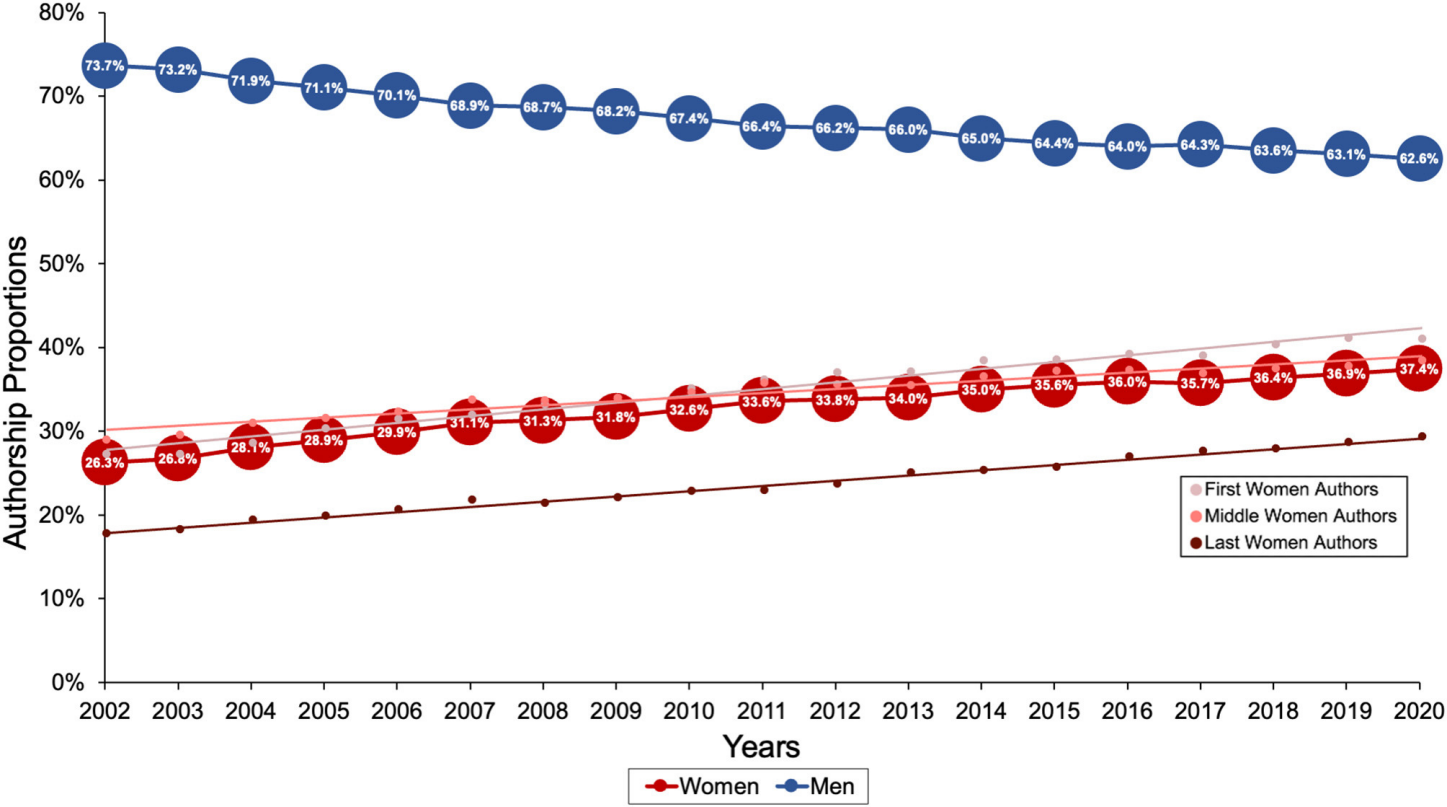
Gender authorship proportion from 2002 up to 2020.

After the NLM reinvention, between 2002 and 2020, 34.2% [567,010/1,656,487 (8,741–76,876/year)] of authors were women (Figure 3). Women occupied 36.4% (104,520/287,324) of first author positions, 35.9% (399,284/1,113,426) of middle author positions and 24.7% (63,206/255,737) of last author positions. During this period, proportion of women authors increased from 26.3 to 37.4% (overall: Spearman rho = 1.00, Cuzick *P* < 0.001; first author: Spearman rho = 1.00, *P* < 0.001; middle author: Spearman rho = 0.99, *P* < 0.001; last author: Spearman rho = 1.00, *P* < 0.001; Figure 3).

### Gender Distribution per Journal Categories

**Figure 4:**
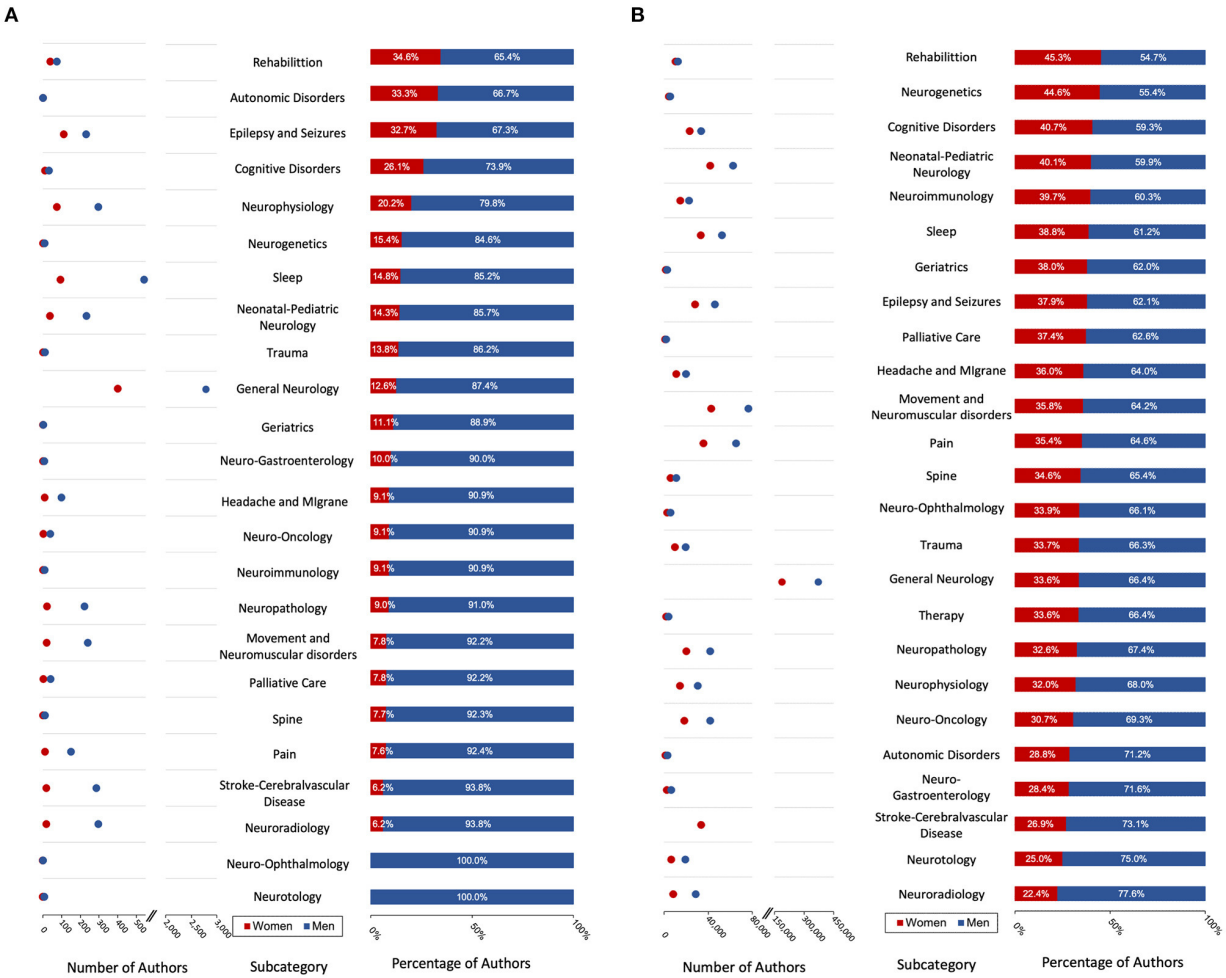
Gender authorship distribution by journal categories. **(A)** From 1946 to 2001 and **(B)** From 2002 to 2020. Left points indicate number of authors in each category. Bars indicate proportion. Categories are ordered by proportion of women authors.

Between 1946 and 2001, no journal category exceeded 637 authors, with the exception of general neurology with a total of 3,187 authors. There were no categories that exceeded 35.0% of women author proportions, ranging from rehabilitation (34.6%) to neuro-ophthalmology and neurotology (0.0%) and different between categories (Chi-Squared test, *P* < 0.001; Figure 4A). Out of 24 journal categories, 12 journal categories did not exceed 10.0% of women proportions.

From 2002 to 2020, all journal categories had more than 3,450 total authors each. Unlike the dataset before the NLM reinvention (1946–2001), no category exceeded 46.0% of women proportions, ranging from rehabilitation (45.3%) to neuroradiology (22.4%) and different between categories (Chi-Squared test, *P* < 0.001; Figure 4B). Five categories had women proportions <30.0% over this time period: neuroradiology (22.4%), neurotology (25.0%), stroke-cerebrovascular disease (26.9%), neuro-gastroenterology (28.4%), and autonomic disorders (28.8%).

### Women Authorship Proportions and Journal-Level Metrics

**Figure 5:**
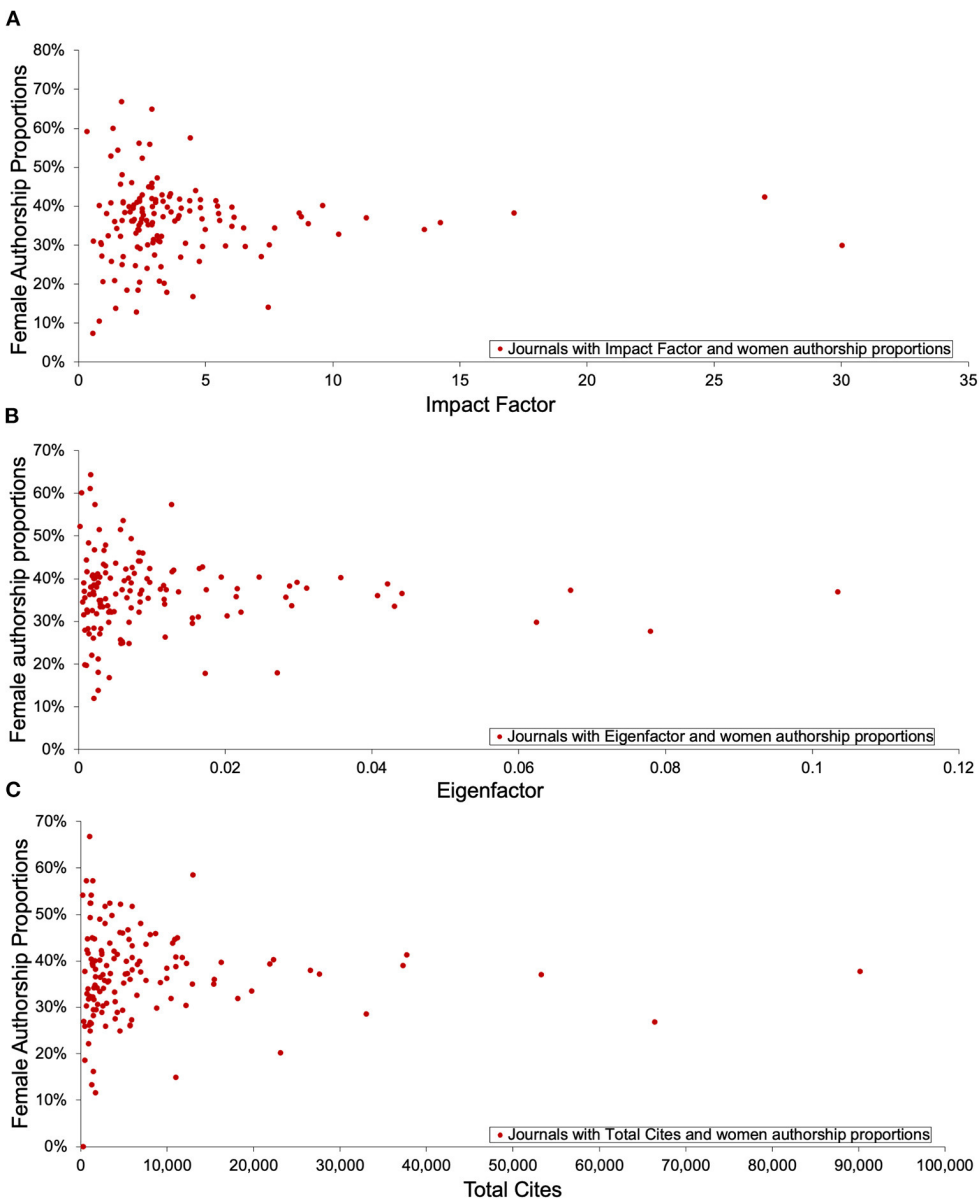
Correlations between author proportions and journal indicator. **(A)** Correlation between Journal Impact Factor (from JCR 2019) and associated women author proportions (2017–2018 data) per journal, **(B)** Correlation between Eigenfactor Score (from JCR 2019) and women author proportions (2015–2019 data) in each journal, **(C)** Correlation between Total Cites (from JCR 2019) and women author proportions (2019 data) in each journal.

There was no correlation observed between the 2019 Impact Factor (IF) and the proportion of women authors in the preceding 2 years (Spearman rho = 0.013, skew = 4.0, kurtosis = 20.7) (Figure 5A). No correlation was observed between the 2019 Eigenfactor and the women author proportion in each journal in the preceding 5 years (Spearman rho = 0.010, skew = 3.3, kurtosis = 13.5) (Figure 5B), nor between the 2019 Total Cites and the women author proportions in 2019 (Spearman rho = 0.076, skew = 4.1, kurtosis = 21.5) (Figure 5C).

### Gender Association Within Publication

**Figure 6:**
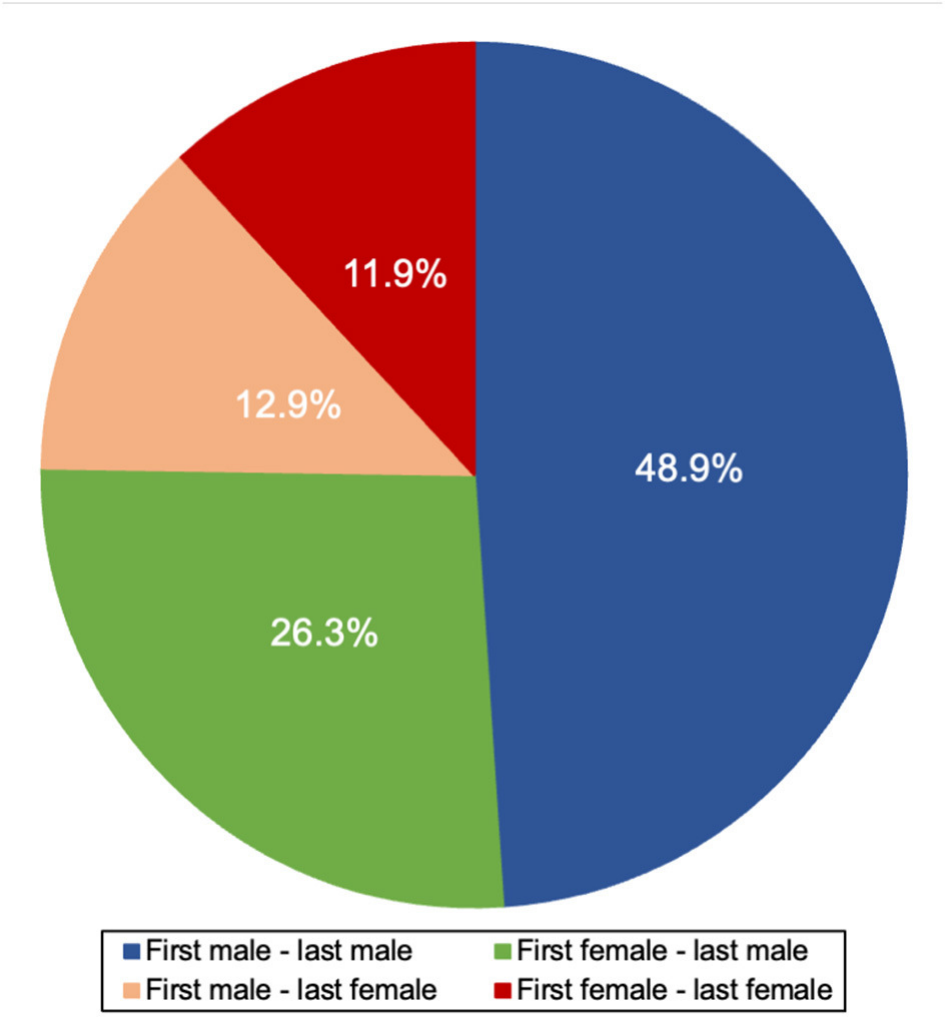
First and last author collaboration proportions.

From 1946 to 2020, 81.5% (*n* = 244,326) of publications had both a first and a last author with an assigned gender. Among these publications, the gender of the fist author was associated with the gender of the last author (McNemar, *P* < 0.001). 48.9% (*n* = 119,566) of publications had a man first author and man last author, 26.3% (*n* = 64,293) had a woman first author and man last author, 12.9% (*n* = 31,462) had a man first author and a woman last author, and 11.9% (*n* = 29,005) had a woman first author and a woman last author. Both men and women last authors were more likely to publish with a man first author, though this was more pronounced for men last authors [odds ratio (men vs. women first author) 1.86 men last author vs. 1.08 women last author] (Figure 6).

## Discussion

This study contributes to the broader literature on authorship gender in neurology and related fields. A study by Dubey et al. examining authorship trends in neuroscience literature reported 29.0% and 17.8% women first and last authors, respectively, in Nature, Neuroscience, and Neuron (19). Our international sample result mirrors that of Pakpoor's et al. (8) analysis of women authorship in three American neurology journals. Unfortunately, the proportions of women authors in all of these studies continue to fall short of the proportion of women neurology residents, which is nearing parity (45.0%). (2). This holds true even when the gender of the first author is only considered. By the single metric of authorship, this suggests that women are proportionately achieving less. Perhaps more concerning is the subtle message sent to those entering the field that scientific publication, particularly in the last author position, is dominated by men. While gender bias is a factor in creating prejudice in the system, there are other elements, such as ethnic and racial prejudice that also account for the lack of diversity in the field of neurology (20).

In medicine, last (senior) authors tend to be of higher rank at their institution (21). Among American full-time medical faculty in all fields in 2018, women proportion varied greatly according to seniority, with 58.0% of instructors being women and only 25.0% of full professors being women (2). Our finding that <25.0% of last authors are women in neurology journals illustrates disparity at senior levels, and demonstrates the disproportionate influence of men in neurology given that last authors typically select the project, act as supervisors, and guide co-authors' participation (22). There could also be bias in the selection process of publications as women are underrepresented among the editorial board members in neurology journals, especially among editors in chief (23). This could potentially create an obstacle for women authors to publish, and thus widening the gender gap in addition to the disparity in authorship positions of women authors in neurology journals. Our finding of men last authors being more likely to publish with men first authors, while women last authors are not more likely to publish with women first authors, indicates how last authors could be (inadvertently) biased in their collaborations and suggests a possible mechanism that contributes to a disparity in women first author involvement (22).

Journal classification provided insight on authorship gender disparity for each subspecialty. Journals classified under neuroradiology showed the largest gender disparity. The results may tie in with the fact that neuroradiology is known to be a men dominated subspecialty similar to the field of radiology in which underrepresentation of women is observed on a global scale (24), with one study reporting women radiologist proportions of 33.5% (25), and on editorial boards based on authorship gender proportions (26). Previous studies have identified potential barriers faced by women radiologists which were common among other fields such as the lack of mentorship, funding, and research opportunities (24, 27, 28). In contrast, the neurogenetics field showed the highest proportion of women authorship. A close look at gender disparity has yet to be done in neurogenetics, though such study may be helpful as a comparator to identify features and attitudes associated with relatively less disparity in authorship gender.

A lack of correlation between journal-level metrics and the gender distribution in our dataset suggests that neurology journals do not display consistent authorship gender bias associated with their impact. This is in contrast to other studies which showed negative correlations between journal Impact Factor and proportion of women authors, and concluded that this may be the result of bias in rejection of author initiated papers and bias in invited authors (29). However, other studies have found results similar to ours in which the journal Impact Factor did not correlate with author gender proportions (30). While it is reassuring that there is not more systemic bias in the high impact journals, it remains concerning that the women authorship proportions in neurology journals vary widely. Possible causes for this include bias specific to individual journals such as gender bias in manuscript submission and/or review. The latter can be addressed with double-blinded reviews, which are associated with increased women authorship without sacrifice of quality (31–34).

The main study limitation is reliance on automated gender assignment, resulting in misclassification bias and excluding some data points for which gender could not be assigned. While self-identification would have confirmed gender outside of Gender-API's binary gender attributions and improved the accuracy of the results, this would have been impractical. We acknowledge that there are many factors that contribute to disparities in scientific authorship that our methodology was unable to assess. One database (PubMed) was used to extract data related to the selected journals and thus, the study results depend on PubMed's indexing system including systemic changes which resulted in many more publications during more recent years. While our methodology collected a large sample of the neurology literature, and we interpreted it in the context of gender proportions in the field of neurology, it is not a sample of publications by neurologists. It is likely that some authors included in this study are not neurologists and our study did not include neurologist publications outside the selected journals. It can also be the case where authors published in neurological journals are not neurologists, many neurologists practice as health care providers and not as researchers, and they may publish in journals that are not necessarily in neurology or specialty-specific journals. Thus, the dataset collected does not encompass the entire population of neurologists. Furthermore, the comparison data for neurology practitioner gender does not reflect the international population as only data from the United States was used. This is due to the fact that it is the largest available dataset regarding gender of neurologists. Moreover, this study did not account for the age of the authors in the analysis, which could potentially alter the trend observed in the correlation of last author-first author association. Finally, scientific authorship is but one marker of productivity and contribution both for a field and for individuals. However, it is an important one due to its prominent role in academic promotion and broad visibility.

The overall trend of women neurology authorship underrepresentation likely parallels women neurologist underrepresentation due to bias that has persisted throughout the years (35). The goal of achieving gender equity not only in presence, but in participation, broadens perspective to enhance research topics, methods and publications. Recruiting and training highly competent scholars is important for research activities in any field, and factors that bias the knowledgeable candidate pool toward one gender at any level hinder productivity of the field and restrict scientific mindsets on given problems (36). Thus, fostering diversity is crucial for growth in both clinical and academic environments (10). This study aims to analyze neurology authorship gender disparities to further understand how to develop solutions to increase women participation in neurology authorship. While authorship is directly related to the number of women neurologists, it is a multifaceted societal issue that must be analyzed from many angles including consideration of systemic bias and gender stereotypes influencing actions by potential authors, reviewers and editors. Promoting women authors as role models in neurology (e.g., through social media and other common platforms) may help to shift this bias. Promoting mentorship among women neurologists could increase their representation in the field and consequently encourage more women to consider pursuing neurology-related careers and become contributors to the scientific literature. Further studies analyzing disparities in neurology are important to fully understand the cause and effect of the existing disparity, to increase women participation, and to accelerate growth of women authorship.

## Conclusion

While the number of women neurologists has increased significantly in the last two decades, the representation of women authors in the neurology literature has probably not caught up. This disparity is even more evident in last authorship position compared to first and middle authorship positions. There is a correlation between first and last authors' gender and the disparity in first author gender is greater among manuscripts with men last authors. Gender authorship disparity exists across all subspecialties but does not correlate with journal impact. These results have important implications that reflect potential causes for reduced achievement of women in neurology. Future studies should investigate factors contributing to authorship disparity including bias that exists in the field of neurology and within the scientific community.

## Data Availability Statement

The raw data supporting the conclusions of this article will be made available by the authors, without undue reservation.

## Author Contributions

AN and AW: had full access to all the data in the study and take responsibility for the integrity of the data and the accuracy of the data analysis. AN: conceptualization, formal analysis, funding acquisition, investigation, methodology, project administration, resources, supervision, validation, visualization, and writing original draft. LY: investigation, visualization, and writing original draft. AL: formal analysis and writing—original draft. X-VT and JK: data curation, software, writing—review, and editing. HM: funding acquisition, validation, and writing—review and editing. AW: funding acquisition, project administration, resources, supervision, validation, writing—review, and editing. All authors contributed to the article and approved the submitted version.

## Conflict of Interest

HM reports financial disclosures from Verily, 2020 therapeutics, Verana Health and Legal firms for consulting, and from Ology Education, American Academy of Neurology, and Vindico CME for speaking and teaching arrangements. Additionally, she reports grants from National Institutes of Health, Myelin Repair Foundation, North American Neuro-Ophthalmology Society, and Stanford. She served on a scientific advisory board for Genentech. The remaining authors declare that the research was conducted in the absence of any commercial or financial relationships that could be construed as a potential conflict of interest.

## Publisher's Note

All claims expressed in this article are solely those of the authors and do not necessarily represent those of their affiliated organizations, or those of the publisher, the editors and the reviewers. Any product that may be evaluated in this article, or claim that may be made by its manufacturer, is not guaranteed or endorsed by the publisher.
